# Time course of hospitalizations in patients with heart failure and chronic obstructive pulmonary disease around sleep-disordered-breathing diagnosis

**DOI:** 10.1007/s11325-024-03242-7

**Published:** 2025-01-15

**Authors:** Maria Tafelmeier, Maximilian Malfertheiner, Florian Zeman, Thomas Penzel, Christoph Schoebel, Winfried Randerath, Marcel Treml, Gary Lotz, Jean-Louis Pepin, Michael Arzt

**Affiliations:** 1 Department of Internal Medicine II (Cardiology, Pneumology, and Intensive Care), University Medical Centre Regensburg, Regensburg, Germany; 2 Center of Pneumology, Hospital Donaustauf, Donaustauf, Germany; 3 Centre for Clinical Studies, University Medical Centre Regensburg, Regensburg, Germany; 4 Sleep Medicine Center, Charité-Universitätsmedizin Berlin, Berlin, Germany; 5 Universitätsmedizin Essen, Ruhrlandklinik - Westdeutsches Lungenzentrum, Essen, Germany; 6 Bethanien Hospital GmbH Solingen, Solingen, Germany; 7 Philips, Clinical and Medical Affairs, Murrysville, USA; 8 Univ. Grenoble Alpes, INSERM, CHU Grenoble Alpes, HP2, Grenoble, France

**Keywords:** COPD, Sleep apnea, Overlap syndrome, Inpatient treatment, Outcome

## Abstract

**Purpose:**

In heart failure (HF) and chronic obstructive pulmonary disease (COPD) populations, sleep-disordered breathing (SDB) is associated with impaired health outcomes. We evaluated whether in patients with HF, concomitant HF and COPD or COPD, the number of hospitalizations would be reduced in the year after testing for SDB with and without treatment initiation compared to the year before.

**Methods:**

We performed a multicentre retrospective study of 390 consecutive sleep-clinic patients who had a primary diagnosis of chronic HF, HF and COPD or COPD and a secondary diagnosis of SDB. The date of SDB-testing was defined as the index date. Data on healthcare utilization was extracted for the 12-month period prior to and after this date.

**Results:**

The initiation of adaptive servoventilation (ASV) and non-invasive ventilation (NIV) treatment resulted in a statistically significant reduction in the number of hospitalisations. While continuous positive airway pressure (CPAP) treatment also demonstrated a reduction in hospitalisations, the observed effect did not reach the level of statistical significance. After accounting for demographics and comorbidities in multivariable regression analyses, only NIV was significantly associated with a reduction in hospitalizations, while CPAP or ASV were not. NIV appears to be underutilized in COPD.

**Conclusions:**

Our data indicate, that patients with HF or COPD and concomitant SDB may benefit from the initiation of appropriate PAP-therapy. Whether treating SDB in HF- and COPD-patients influences healthcare utilization merits further investigation.

**Supplementary Information:**

The online version contains supplementary material available at 10.1007/s11325-024-03242-7.

## Introduction

Sleep-disordered breathing (SDB), including obstructive and central sleep apnoea (OSA and CSA), and chronic hypercapnic respiratory failure are increasingly recognized as modulators of trajectories of various chronic disorders, such as heart failure (HF) [1], and chronic obstructive pulmonary disease (COPD) [2]. Due to shared risk factors and most likely a bi-directional causal relationship, approximately half of the patients with chronic HF are diagnosed with SDB [3]. In COPD, OSA (overlap syndrome) and chronic hypercapnic respiratory failure occur in 10–20% [4] and 25% [5] of patients, respectively.

HF and COPD exacerbations negatively influence patients’ quality of life, functioning, and survival and are a common cause for medical consultations and hospitalizations [1, 2]. Remarkably, the presence of SDB has been described as a significant predictor of higher healthcare utilization and mortality in HF and COPD patients [1]. The objective of this study was to evaluate whether the initiation of SDB treatment with CPAP, ASV or NIV in patients with HF, concomitant HF and COPD or COPD was associated with a reduced risk of hospitalisations.

## Methods

This retrospective analysis was performed using de-identified medical data collected in four participating European sleep centres in France and Germany. The study was approved by each institution’s Research Ethics Committee.

Medical records were reviewed to identify patients aged 21–85 years who had undergone polysomnography (PSG) or polygraphy (PG) between January 2012 and February 2017. The records were selected based on the following criteria:

- Patients between 21 and 85 years.
- Primary diagnosis of chronic HF, HF and COPD (HF + COPD) or COPD.
- Secondary diagnosis of SDB (OSA, CSA with or without chronic hypercapnic respiratory failure).

The severity of SDB was assessed using the apnoea-hypopnea index (AHI). An AHI of ≥ 15/h was considered the cut-off for the diagnosis of moderate SDB; patients with SDB and ≥ 50% central apneas were classified into the CSA group and patients with < 50% central apneas into the OSA group [3]. The date of SDB-testing (PSG or PG) was defined as the index date. Chronic hypercapnic respiratory failure was defined as a PaCO_2_ level exceeding 45 mmHg. Data on healthcare utilization was extracted for the 12-month period prior to and after this date. Hospitalizations for HF or COPD and mortality were identified by reviewing medical records or being self-reported by patients through standardised phone interviews and questionnaires. For details on statistical analyses, please refer to the online data supplement.

The data that support the findings of this study are available from the corresponding author upon reasonable request.

## Results

Among the 390 elderly and predominantly male sleep-clinic patients of this analysis, 68% were diagnosed with HF, 16% with HF + COPD and 17% with COPD (Table 1). While patients with HF and HF + COPD had a median AHI that indicated a moderate degree of OSA or CSA, patients with COPD had a significantly lower median AHI corresponding to a mild degree of OSA or CSA (Table 1).

**Table 1:** Baseline characteristics of the patients in the study

	Total sample	heart failure	heart failure and COPD	COPD	*p*-value
**Demographic data**					
n (%)	390 (100)	264 (68)	61 (16)	65 (17)	
Age, years	68 ± 11	69 ± 11^c^	68 ± 10	64 ± 9^c^	**0.001** ^**A**^
Male sex, n (%)	287 (74)	200 (76)	47 (77)	40 (62)	0.053 ^Chi^
Body mass index, kg/m²	30.3 ± 7.5	30.2 ± 7.5	32.3 ± 7.1 ^b^	28.8 ± 7.4 ^b^	**0.035** ^**A**^
Obesity, n (%)	153 (40)	93 (36) ^a^	36 (59) ^a b^	24 (38) ^b^	**0.003** ^**Chi**^
**Comorbidities**					
NYHA III/IV, n (%)	124 (44)	97 (42)	27 (53)	-	0.523 ^Chi^
LV ejection fraction, %	44 ± 14	43 ± 14	46 ± 14	-	0.259 ^A^
LV ejection fraction ≤ 45%, n (%)	125 (54)	104 (56)	21 (46)	-	0.211 ^Chi^
Ischemic heart failure, n (%)	204 (69)	172 (69)	32 (64)	-	0.457 ^Chi^
Coronary artery disease, n (%)	199 (51)	153 (58) ^c^	36 (59) ^b^	10 (16) ^b c^	**< 0.001** ^Chi^
Atrial fibrillation, n (%)	132 (34)	107 (41) ^c^	19 (32) ^b^	6 (9) ^b c^	**< 0.001** ^Chi^
History of stroke, n (%)	31 (8)	25 (10)	2 (3)	4 (6)	0.492 ^Chi^
Renal failure, n (%)	105 (27)	81 (31) ^c^	22 (37) ^b^	2 (3) ^b c^	**< 0.001** ^**Chi**^
History of alcohol abuse, n (%)	60 (38)	27 (33)	10 (39)	23 (45)	0.398 ^Chi^
**Pulmonary function diagnostics**					
Vital capacity, % of predicted	80.0 ± 21.3	-	81.1 ± 20.1	79.3 ± 22.2	0.679 ^A^
FEV_1_, % of predicted	59.3 ± 19.7	-	63.1 ± 17.3	57.0 ± 20.9	0.135 ^A^
Total lung capacity, % of predicted	106.8 ± 23.9	-	98.8 ± 26.4	111.9 ± 21.0	**0.009** ^A^
**Daytime arteriocapillary blood gas analysis**					
pH	7.43 (7.41; 7.45)	7.43 (7.40; 7.45)	7.42 (7.40; 7.45)	7.43 (7.41; 7.46)	0.806 ^KW^
PaO_2_, mmHg	72.0 (63.4; 82.3)	77.4 (67.6; 89.5) ^c^	68.5 (62.5; 81.4) ^b^	65.8 (57.1; 73.4) ^b c^	**< 0.001** ^**KW**^
PaCO_2_, mmHg	38.0 (35.5; 41.2)	37.3 (35.3; 40.3) ^c^	38.3 (35.5; 41.8)	39.7 (37.1; 44.6) ^c^	**< 0.001** ^**KW**^
PaCO_2_ > 45 mmHg, n (%)	28 (8)	10 (4) ^c^	5 (9)	13 (21) ^c^	**< 0.001** ^**Chi**^
HCO_3-_, mmHg	25.0 (23.6; 26.5)	24.5 (23.4; 25.8) ^c^	25.3 (23.3; 26.4) ^b^	26.7 (24.9; 28.0) ^b c^	**< 0.001** ^**KW**^
Base excess, mmol/l	1.15 (-0.46; 2.67)	0.69 (-0.53; 2.20) c	1.07 (-1.02; 2.70) ^b^	2.66 (0.40; 3.57) ^b c^	**0.002** ^**KW**^
**Nocturnal respiration data**					
Apnea hypopnoea index, per hour	25 (12; 45)	29 (16; 47) ^c^	25 (13; 46) ^b^	13 (6; 29) ^b c^	**< 0.001** ^**KW**^
Apnea hypopnea index ≥ 15/h, n (%)	272 (70)	199 (76) ^c^	44 (73) ^b^	29 (45) ^b c^	**< 0.001** ^**Chi**^
Central apnea index-apnea index ratio, %	49 (41; 53)	50 (40; 55)	48 (37; 51)	49 (44; 50)	0.129 ^KW^
Oxygen desaturation index, per hour	28 (12; 44)	28 (13; 45) ^c^	28 (15; 52) ^b^	14 (5; 31) ^b c^	**0.001** ^**KW**^
Mean SpO_2_, %	93.0 (91.0; 94.0)	93.0 (92.0; 94.0) ^a c^	92.0 (90.0; 94.0) ^a^	91,0 (89.7; 94.0) ^c^	**< 0.001** ^**KW**^
Time of SpO_2_ < 90%/total recording time	22 (4; 78)	19 (4; 56)	36 (4; 150)	26 (3; 162)	0.154 ^KW^
Total recording time, min	446 (410; 478)	447 (411; 478)	445 (408; 482)	438 (399; 473)	0.867 ^KW^
Total sleep time, min	323 (265; 370)	328 (275; 372)	314 (256; 366)	319 (247; 369)	0.598 ^KW^
Arousal index, per hour	24 (5; 41)	25 (6; 42)	25 (2; 36)	19 (4; 40)	0.494 ^KW^
**Treatment initiation**					
no treatment	137 (35)	84 (32) ^c^	19 (32) ^b^	34 (52) ^b c^	**0.008** ^**Chi**^
continuous positive airway pressure	146 (37)	103 (40)	18 (31)	25 (39)	0.228 ^Chi^
adaptive servoventilation	62 (16)	50 (19) ^c^	12 (20) ^b^	0 (0) ^b c^	**< 0.001** ^**Chi**^
non-invasive ventilation	33 (9)	18 (7)	10 (17)	5 (8)	0.053 ^Chi^
mandibular assist device	3 (1)	2 (1)	0 (0)	1 (1)	0.618 ^Chi^
mandibular repositioning osteotomy	2 (0)	2(1)	0 (0)	0 (0)	0.619 ^Chi^

Data are presented as mean ± standard deviation, as median (25.; 75. percentile) or as absolute and relative frequencies. ^A^ ANOVA; ^Chi^ Chi-square test; ^KW^ Kruskal-Wallis test. ^a^ p_heart failure vs. heart failure and COPD_ <0.05; ^b^ p_heart failure and COPD vs. COPD_ <0.05; ^c^ p_heart failure vs. COPD_ <0.05. COPD: chronic obstructive pulmonary disease; NYHA: New York Heart Association; LV: left ventricular; FEV_1_: forced expiratory volume at 1 s. Polysomnography: *n* = 359, polygraphy: *n* = 31. Adaptive servoventilation was used outside the intended indication in 18 patients

**Fig. 1:**
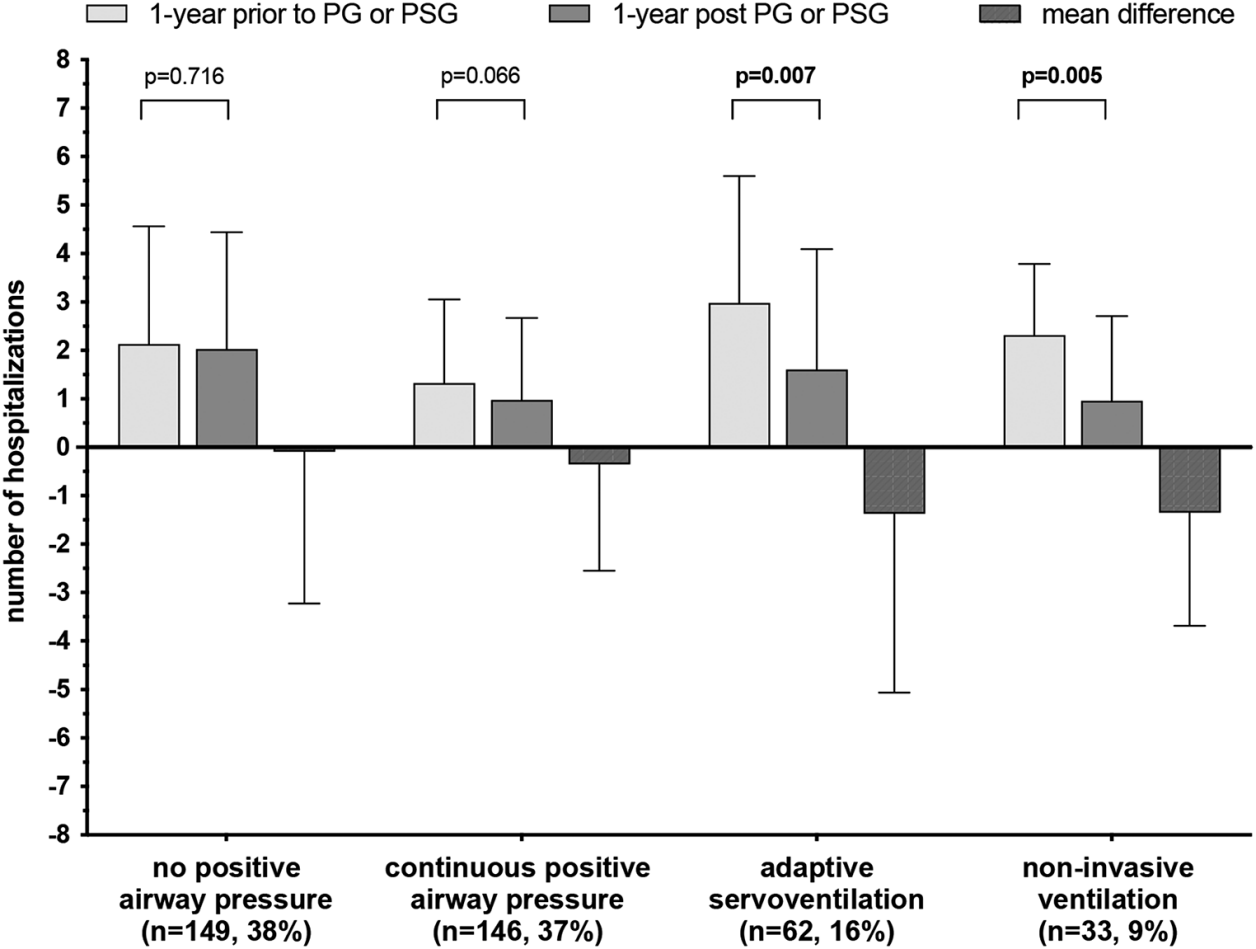
Hospitalizations prior and post assessment of SDB. Number of hospitalizations and mean difference in the number of hospitalizations between the year prior and the year after assessment of SDB using polysomnography or polygraphy in patients without positive airway pressure, with continuous positive airway pressure, with adaptive servoventilation, and with non-invasive ventilation. Data are presented as mean ± standard deviation. PG: polygraphy; PSG: polysomnography

The initiation of ASV and NIV treatment resulted in a statistically significant reduction in the number of hospitalizations (Fig. 1). While CPAP treatment also demonstrated a reduction in hospitalizations, the observed effect did not reach the level of statistical significance (Fig. 1). The hospitalization rate remained unchanged in the absence of PAP treatment (Fig. 1). After accounting for demographic variables and comorbidities in multivariable regression analyses, only NIV-therapy was significantly associated with a reduction in hospitalizations, whereas CPAP and ASV were not (Table 2).

**Table 2:** Predictors of hospitalizations after testing for sleep-disordered breathing– multivariable regression analysis with reduction in hospitalizations as dependent variable

Independent variable	Multivariable regression analysis (CPAP-model)			Multivariable regression analysis (ASV-model)			Multivariable regression analysis (NIV-model)			Multivariable regression analysis (all PAP modalities-model)		
	B	95% CI	*p*-value	B	B	B	B	95% CI	*p*-value	B	95% CI	*p*-value
Age, years	1.007	(0.980; 1.035)	0.625	1.021	(0.989; 1.053)	0.199	1.023	(0.987; 1.060)	0.207	1.018	(0.994; 1.043)	0.139
Male sex	0.554	(0.291; 1.056)	0.073	0.540	(0.237; 1.229)	0.142	0.352	(0.145; 0.853)	**0.021**	0.584	(0.332; 1.026)	0.061
Body mass index, kg/m²	1.032	(0.988; 1.078)	0.157	1.013	(0.954; 1.077)	0.668	1.024	(0.963; 1.089)	0.446	1.018	(0.982; 1.055)	0.337
Apnea hypopnea index, per hour	0.995	(0.976; 1.014)	0.580	0.993	(0.974; 1.012)	0.467	0.996	(0.975; 1.017)	0.701	0.996	(0.981; 1.011)	0.625
Heart failure	0.457	(0.223; 0.939)	**0.033**	0.524	(0.209; 1.314)	0.168	0.376	(0.150; 0.941)	**0.037**	0.365	(0.182; 0.733)	**0.005**
Continuous positive airway pressure (reference: no PAP-treatment)	1.313	(0.735; 2.347)	0.358									
Adaptive servoventilation (reference: no PAP-treatment)				0.763	(0.349; 1.667)	0.497						
Non-invasive ventilation (reference: no PAP-treatment)							0.259	(0.083; 0.809)	**0.020**			
Positive airway pressure (all modalities)										0.948	(0.568; 1.581)	0.838

Multivariable regression analyses with different PAP-modalities as independent variables (CPAP-model, NIV-model, ASV-model and all PAP-modalities model). Association of predictors of a reduction in the number of hospitalizations after testing for sleep-disordered breathing. Values are presented as B: Regression coefficient and 95% CI: confidence interval. PAP: Positive airway pressure; CPAP: continuous positive airway pressure; ASV = adaptive servoventilation; NIV = non-invasive ventilation

The online data supplement provides supplementary baseline characteristics (Table S1), further information on hospitalizations (Table S2), details on the commencement of treatment according to SDB-subgroups (Table S3), and data on the prevalence of SDB and chronic hypercapnic respiratory failure (Table S4). Furthermore, supplementary sensitivity analyses of distinct patient subgroups and with regard to mask-based treatment are presented in Fig. S1 and Fig. S2.

## Discussion

To our knowledge, this is the first study to assess the time course of hospitalizations preceding and following SDB testing and treatment initiation in patients with HF and COPD. Previous studies in HFrEF-patients that evaluated hospitalizations and mortality after SDB-testing found a significant association of PAP-treatment and improved survival either compared to those, who were not tested for SDB [6], or those, who refused PAP-therapy [7]. The key differences of the present analysis to such studies are (1) that we investigated hospitalizations rather than mortality, (2) that the time course of hospitalizations before and after SDB-testing was studied, and (3) compared between different diseases (HF, HF + COPD and COPD). The present analysis revealed a notable decline in hospitalizations following the commencement of PAP therapy, whereas the incidence of hospitalization remained unaltered in the absence of PAP treatment. While the level of statistical significance was not reached in comparison to ASV or NIV therapy, CPAP therapy also demonstrated a noteworthy effect in reducing hospitalizations. The initiation of PAP treatment was observed to be most efficacious in patients with HF (including HFrEF and HFpEF, and irrespective of etiology and NYHA classification), as well as in those with OSA. Our findings are divergent from those of previous studies. Prior meta-analyses have indicated that CPAP and ASV use do not confer benefits with respect to cardiovascular outcomes and mortality [8]. While the ADVENT HF trial demonstrated that ASV can be safely used in patients with HFrEF and sleep apnoea without increasing the risk of mortality and may enhance quality of life for specific patient populations, the evidence does not substantiate a notable reduction in hospitalizations or overall mortality [9].

Alongside an overall deterioration of the patients´ health condition and quality of life, COPD progression results in frequent physician visits and hospitalizations. With one third of COPD-patients being admitted for COPD exacerbation in the year after testing for SDB, disease progression may also have contributed to the detected increase in hospitalization rate in our cohort. After accounting for demographics and comorbidities in multivariable regression analyses, only NIV was significantly associated with a reduction in hospitalizations, while CPAP or ASV were not. Although NIV may have little impact on sleep quality, it was shown to improve overall quality of life, improve functional capacity and reduce hospital admissions and mortality in patients with chronic hypercapnic COPD [10, 11]. In the present study, NIV appears to be underutilized in COPD-patients, since the proportion of patients with daytime hypercapnia (21%) and the expected rate of chronic hypercapnic respiratory failure (25%)^5^ are higher compared to the frequency of NIV-users in the COPD-group (8%).

The retrospective design and the relatively small sample size of our study may limit the generalizability of the findings. We cannot provide detailed information regarding the initial diagnosis of heart failure or COPD as well as the precise dates of hospitalization or the length of time spent in hospital. Furthermore, the reason for a considerable number of hospitalizations could not be determined due to a lack of available information. Given the limited sample size, the results of the multivariable regression models should be interpreted with caution.

Although our findings are promising and indicate that PAP treatment may have the potential to reduce hospitalizations, further large-scale prospective studies are necessary to identify specific patient populations that may benefit from the initiation of adequate PAP therapy.

## Electronic supplementary material

Below is the link to the electronic supplementary material.

Supplementary Material 1

Supplementary Material 2

## Data Availability

The data that support the findings of this study are available from the corresponding author upon reasonable request.
