# Supplementary material for: Time course of hospitalizations in patients with heart failure and chronic obstructive pulmonary disease around sleep-disordered-breathing diagnosis
Source: Sleep Breath. 2025 Jan 15;29(1):79. doi: 10.1007/s11325-024-03242-7 (PMC11735569; doi:10.1007/s11325-024-03242-7)
Supplement: Supplementary file 1 — Supplementary Material 1 [file 11325_2024_3242_MOESM1_ESM.docx]

**Time course of hospitalizations in patients with heart failure and chronic obstructive pulmonary disease around sleep-disordered-breathing diagnosis**

**- Online data supplement -**

**Authors**:

Maria Tafelmeier^1^, Maximilian Malfertheiner^2^, Florian Zeman^3^, Thomas Penzel^4^, Christoph Schoebel^5^, Winfried Randerath^6^, Marcel Treml^6^, Gary Lotz^7^, Jean-Louis Pepin^8^, Michael Arzt^1^

**Author affiliations:**

1. Department of Internal Medicine II (Cardiology, Pneumology, and Intensive Care), University Medical Centre Regensburg, Regensburg, Germany
2. Center of Pneumology, Hospital Donaustauf, Donaustauf, Germany

^3^ Centre for Clinical Studies, University Medical Centre Regensburg, Regensburg, Germany

^4^ Charité - Universitätsmedizin Berlin, Berlin, Germany

^5^ Ruhrlandklinik, Essen, Germany

^6^ Bethanien Hospital GmbH Solingen, Solingen, Germany

^7^ Philips, Clinical and Medical Affairs, Murrysville, USA

^8^ Univ. Grenoble Alpes, INSERM, CHU Grenoble Alpes, HP2, Grenoble, France

**Supplemental methods**

**Statistical analysis**

Statistical analyses were done with SPSS 26.0 (IBM, New York, USA). Data are presented as mean ± standard deviation for normally distributed data and median (25.;75. percentile) for non-normally distributed data; categorical variables are described as absolute and relative frequencies. Differences between groups were compared using ANOVA for normally distributed continuous variables, the Kruskal-Wallis test for non-normally distributed continuous variables, and the Person’s chi-square test of independence for categorical variables. A multivariable linear regression model with key demographic parameters and clinically relevant confounders was calculated. While this main multivariable linear regression model was adjusted for all PAP-modalities, three supplementary multivariable linear regression models were used for sensitivity analyses that were adjusted for CPAP, NIV or ASV only. Regression coefficient (B) with corresponding 95%-confidence interval (CI) is presented as effect estimate for each predictor. A two-sided p-value of ≤0.05 was considered statistically significant for all analyses.

**Supplemental results**

Please refer to Table S2 for details on the reasons for hospitalization after testing for sleep-disordered breathing.

Most frequently, 17% and 13% of patients with HF and HF+COPD were hospitalized for HF decompensation (Table S2). One third of COPD-patients being admitted for COPD exacerbation (Table S2).

Changes in hospitalization rates after SDB-testing according to HF and COPD (Figure S1) were not significantly altered by SDB-status (no SDB, OSA or CSA; Fig. S1A) or in HF-patients by left ventricular ejection fraction (≤45 or >45%; Fig. S1B) or NYHA-class (NYHA-class i/II or NYHA-class III/IV; Fig. S1D). Among patients with HF, the reduction in hospitalizations was higher in those with non-ischemic HF compared to those with ischemic HF (Fig. S1C). Furthermore, supplementary sensitivity analyses with regard to mask-based treatment are presented in Fig. S2.

**Supplementary tables**

Table S1 Supplementary baseline characteristics of the patients in the study

|  | **total sample** | **heart failure** | **heart failure and COPD** | **COPD** | **p-value** |
| --- | --- | --- | --- | --- | --- |
| **Cardiovascular risk factors** | | | | | |
| Hypertension, n (%) | 300 (77) | 206 (78)^c^ | 55 (90)^b^ | 39 (61)^b c^ | **0.003 ^Chi^** |
| Hyperlipidaemia, n (%) | 213 (55) | 158 (60)^c^ | 40 (66)^b^ | 15 (24)^b c^ | **<0.001 ^Chi^** |
| Diabetes mellitus, n (%) | 130 (34) | 95 (36)^c^ | 26 (43)^b^ | 9 (14)^b c^ | **0.007 ^Chi^** |
| Smoking, n (%) | 177 (67) | 86 (54)^a c^ | 40 (91)^a^ | 51 (84)^c^ | **<0.001 ^Chi^** |
| **Symptoms of COPD** | | | | | |
| Frequency of COPD exacerbations ≥2/year, n (%) | 24 (31) | - | 9 (35) | 15 (29) | **0.039 ^Chi^** |
| Moderate to severe COPD symptoms (CAT ≥ 10, mMRC ≥ 2), n (%) | 52 (85) | - | 18 (90) | 34 (83) | 0.075 ^Chi^ |
| **Medication** | | | | | |
| Betablocker, n (%) | 281 (73) | 216 (83)^c^ | 47 (78) ^b^ | 18 (28) ^b c^ | **<0.001 ^Chi^** |
| ACE inhibitor, n (%) | 268 (70) | 200 (77)^c^ | 41 (68) ^b^ | 27 (42) ^b c^ | **<0.001 ^Chi^** |
| Spironolactone, n (%) | 69 (18) | 53 (20) | 12 (20) | 4 (6) | 0.085 ^Chi^ |
| Diuretics, n (%) | 40 (10) | 29 (11) | 8 (13) | 3 (5) | 0.226 ^Chi^ |
| Long-acting β_2_-agonist, n (%) | 57 (15) | 7 (3)^a c^ | 24 (40)^a^ | 26 (40)^c^ | **<0.001 ^Chi^** |
| Long-acting muscarinic antagonist, n (%) | 61 (16) | 6 (2)^a c^ | 27 (45)^a^ | 28 (46)^c^ | **<0.001 ^Chi^** |
| Inhaled corticosteroids, n (%) | 57 (15) | 12 (5)^a c^ | 17 (28)^a^ | 28 (44)^c^ | **<0.001 ^Chi^** |
| Oral corticosteroids, n (%) | 28 (7) | 10 (4)^c^ | 1 (2) | 17 (27)^c^ | **<0.001 ^Chi^** |

Data are presented as absolute and relative frequencies. ^Chi^Chi-square test; ^a^p_heart failure vs. heart failure and COPD_ <0.05; ^b^p_heart failure and COPD vs. COPD_ <0.05; ^c^p_heart failure vs. COPD_<0.05. COPD: chronic obstructive pulmonary disease; ACE: Angiotensin-converting enzyme.

Table S2 Reasons for hospitalization after testing for sleep-disordered breathing

|  | **total sample** | **heart failure** | **heart failure and COPD** | **COPD** | **p-value** |
| --- | --- | --- | --- | --- | --- |
| **Reasons for hospitalization** | | | | | |
| Heart failure decompensation, n (%) | 53 (14) | 44 (17)^c^ | 8 (13)^b^ | 1 (1)^b c^ | **0.006 ^Chi^** |
| Acute cardiovascular event, n (%) | 4 (1) | 2 (1) | 2 (3) | 0 (0) | 0.142 **^Chi^** |
| COPD exacerbation, n (%) | 29 (7) | 1 (0)^a c^ | 6 (10)^a b^ | 22 (34)^b c^ | **<0.001 ^Chi^** |
| other reason, n (%) | 74 (19) | 44 (17) ^c^ | 10 (16) | 20 (31) ^c^ | **0.029 ^Chi^** |

Data are presented as absolute and relative frequencies. ^Chi^Chi-square test; ^a^p_heart failure vs. heart failure and COPD_ <0.05; ^b^p_heart failure and COPD vs. COPD_ <0.05; ^c^p_heart failure vs. COPD_<0.05. COPD: chronic obstructive pulmonary disease.

Table S3 Information on treatment initiation according to different patient subgroups

|  | **Total sample** | **no SDB (AHI <15/h)** | **OSA (AHI ≥15/h)** | **CSA (AHI ≥15/h)** | **p-value** |
| --- | --- | --- | --- | --- | --- |
| **Treatment initiation** | | | | | |
| no treatment | 135 (36) | 72 (63) ^a c^ | 26 (18) ^a b^ | 37 (32) ^b c^ | **<0.001 ^Chi^** |
| continuous positive airway pressure | 137 (36) | 26 (23) ^a^ | 82 (56) ^a b^ | 29 (25) ^b^ | **<0.001 ^Chi^** |
| adaptive servoventilation | 61 (16) | 4 (3) ^a c^ | 18 (12) ^a b^ | 39 (34) ^b c^ | **<0.001 ^Chi^** |
| non-invasive ventilation | 33 (9) | 8 (7) | 19 (13) ^b^ | 6 (5) ^b^ | 0.067 ^Chi^ |
| mandibular assist device | 3 (1) | 3 (3) ^a^ | 0 (0) ^a^ | 0 (0) | **0.031 ^Chi^** |
| mandibular repositioning osteotomy | 2 (0) | 1 (1) | 1 (1) | 0 (0) | 0.627 ^Chi^ |

Data are presented as absolute and relative frequencies. ^Chi^Chi-square test. ^a^p_no SDB vs. OSA_ <0.05; ^b^p_OSA vs. CSA_ <0.05; ^c^p_no SDB vs. CSA_<0.05. SDB: sleep-disordered breathing; AHI: apnea hypopnea index; OSA: obstructive sleep apnea; CSA: central sleep apnea. Polysomnography: n=359, polygraphy: n= 31. Adaptive servoventilation was used outside the intended indication in 18 patients.

Table S4 Information on prevalence numbers for SDB and chronic hypercapnic respiratory failure

|  | **total sample** | **heart failure** | **heart failure and COPD** | **COPD** | **p-value** |
| --- | --- | --- | --- | --- | --- |
|  | | | | | |
| no sleep-disordered breathing (AHI <15/h), n (%) | 115 (30) | 63 (24)^c^ | 16 (28)^b^ | 36 (58)^b c^ | **<0.001 ^Chi^** |
| obstructive sleep apnea (AHI ≥15/h), n (%) | 148 (39) | 101 (39) | 27 (47) | 20 (32) | 0.276 ^Chi^ |
| central sleep apnea (AHI ≥15/h), n (%) | 116 (31) | 95 (37)^c^ | 15 (26) | 6 (10)^c^ | **<0.001 ^Chi^** |
| chronic hypercapnic respiratory failure, n (%) | 28 (8) | 10 (4)^c^ | 5 (9) | 13 (21)^c^ | **<0.001 ^Chi^** |

Data are presented as absolute and relative frequencies. ^Chi^Chi-square test; ^a^p_heart failure vs. heart failure and COPD_ <0.05; ^b^p_heart failure and COPD vs. COPD_ <0.05; ^c^p_heart failure vs. COPD_<0.05. COPD: chronic obstructive pulmonary disease; AHI: apnea hypopnea index.

**Supplementary figure**

**Figure S1:** **Sensitivity analyses - as for figure 1, with different patient subgroups**

Mean difference in the number of hospitalizations between the year prior and the year after assessment of SDB using polysomnography or polygraphy. An AHI of ≥15/h was considered the cut-off for the diagnosis of SDB; patients with SDB and ≥50% central apneas are classified into the CSA group and patients with <50% central apneas into the OSA group. Data are presented as mean ± standard deviation for patients with heart failure ± COPD or COPD (A), patients with left ventricular ejection fraction ≤45% or >45% (B), patients with ischemic or non-ischemic heart failure (C), and patients with NYHA-class I/II or NYHA-class III/IV (D). SDB: sleep-disordered breathing; AHI: apnea hypopnea index; OSA: obstructive sleep apnea; CSA: central sleep apnea; COPD: chronic obstructive pulmonary disease; LVEF: left ventricular ejection fraction; NYHA: New York Heart Association.

**Figure S2:** **Sensitivity analyses - as for figure 1, in patients without and with mask-based treatment**

Mean difference in the number of hospitalizations between the year prior and the year after assessment of SDB using polysomnography or polygraphy in patients without and with mask-based treatment. Data are presented as mean ± standard deviation for patients with heart failure, heart failure and COPD or COPD (A) and for patients without SDB (AHI <15/h), with SDB (AHI ≥15/h), with OSA and with CSA (B). An AHI of ≥15/h was considered the cut-off for the diagnosis of SDB; patients with SDB and ≥50% central apneas are classified into the CSA group and patients with <50% central apneas into the OSA group. COPD: chronic obstructive pulmonary disease; SDB: sleep-disordered breathing; OSA: obstructive sleep apnea; CSA: central sleep apnea; AHI: apnea hypopnea index.

**Fig. S1**

**B**

**A**

**D**

**C**

**Fig. S2**

**A**

**B**
